# Uncovering Latent Structures of School Belonging, Emotional Problems, Psychological Symptoms, Meaningful School, and Ostracism: A Latent Profile Analysis

**DOI:** 10.1002/brb3.71163

**Published:** 2026-02-09

**Authors:** Caner Doğrusever, Hacer Yıldırım Kurtuluş, Alican Kaya, Nuri Türk, Murat Yıldırım

**Affiliations:** ^1^ Department of Guidance and Psychological Counselling Siirt University Siirt Türkiye; ^2^ Department of Guidance and Psychological Counselling Yıldız Technical University İstanbul Türkiye; ^3^ Department of Guidance and Psychological Counselling Ağrı İbrahim Çeçen University Ağrı Türkiye; ^4^ Department of Psychology Faculty of Science and Letters Agri Ibrahim Cecen University Ağrı Türkiye; ^5^ Psychology Research Center Khazar University Baku Azerbaijan

**Keywords:** emotional problems, meaningful school, ostracism, psychological symptoms, school belonging

## Abstract

**Background:**

Despite increasing interest in school belonging, few studies have applied person‐centered approaches to explore how emotional and psychological factors interact within adolescent populations. This study aimed to identify latent profiles of adolescents based on their experiences of school belonging, emotional problems, psychological symptoms, meaningful school engagement, and ostracism.

**Method:**

A multidimensional construct was developed, incorporating indicators such as ignorance, exclusion, somatization, depression, anxiety, purpose enjoyment, responsible understanding, and prosociality. A convenience sample of 749 adolescents (64.4% female; *M_age_* = 15.27, SD = 1.25) was recruited. Latent profile analysis was conducted to uncover distinct student profiles.

**Results:**

Four profiles emerged: (1) Distressed but Included—high emotional problems despite near‐average ostracism; (2) Adaptive and Successful—high meaningful school engagement and prosociality with low emotional problems; (3) Balanced and Typical—normative levels across all indicators; and (4) Ostracized with Psychological Risk—the highest levels of ostracism and emotional problems.

**Conclusion:**

School belonging plays a critical role in identifying psychological risk and resilience among adolescents. Latent profile analysis offers a nuanced framework for developing targeted interventions to support students’ emotional and social well‐being.

## Graphical abstract

This study explored how school belonging interacts with emotional and psychological factors among adolescents. Using latent profile analysis with a sample of 749 students, four distinct profiles were identified. The first group, Distressed but Included, showed high emotional problems despite average levels of ostracism. The second, Adaptive and Successful, demonstrated strong school engagement and prosocial behavior with low emotional problems. The third, Balanced and Typical, reflected normative levels across all indicators. Finally, the Ostracized with Psychological Risk group experienced the highest levels of ostracism alongside severe emotional difficulties. Together, these profiles highlight that school belonging is not uniform; it can signal both resilience and vulnerability. Recognizing these differences provides a framework for targeted interventions that strengthen emotional well‐being and social support in schools

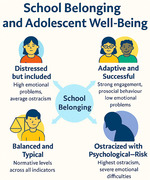

## 1 Introduction

Adolescence is a critical period for individuals' psychosocial adjustment, academic development, well‐being, and mental health (Cengiz et al. 2025; Steinberg and Morris 2001; Yıldırım et al. 2025). During this period, students' school experiences determine not only their academic success but also their social relationships, sense of belonging, and psychological well‐being (Arslan et al. 2022; Wong et al. 2021; Yıldırım et al. 2023). Longitudinal studies conducted in recent years have revealed that school belongingness plays a preventive role in the development of psychological problems such as depression and anxiety in adolescents (Allen et al. 2024). Furthermore, it has been shown that students' positive perception of the school climate is a strong predictor of mental health (Long et al. 2021; Sipahioğlu et al. 2023). In this context, it is important to examine how different psychosocial variables (e.g., social exclusion, psychological symptoms, meaning in school, and prosociality) are related to school belongingness among adolescents. However, in the literature, most of these variables have generally been addressed through independent and variable‐centered analyses. Adolescents, however, can form heterogeneous groups that carry both risk and protective factors. Therefore, our study aims to identify distinct students’ profiles using the latent profile analysis (LPA) and to examine how school belonging affects membership in these profiles.

## 2 Ostracism and School Belonging

One of the most fundamental psychosocial needs of human beings is to connect with others and be accepted. Threats to this need for belonging can be perceived as an existential threat to the individual (Kiuru et al. 2024). Unfortunately, a significant proportion of adolescents (approximately 10%–20%) consistently feel lonely and socially excluded (Lyyra et al. 2022). Ostracization is defined in the literature as a social stressor that arises when an individual is excluded from or ignored in social relationships (Williams 2001). During adolescence, this experience is a social risk factor and is frequently observed in both peer relationships and school settings (Baumeister and Leary 1995). Ostracization directly undermines adolescents' need for belonging and acceptance, weakening their attachment to school and leading to social isolation (Büyükcebeci and Deniz 2017; Tan et al. 2025).

Recent studies show that ostracism has powerful effects on adolescents' psychological health and sense of belonging (Arslan and Yıldırım 2022; Turan et al. 2024). Arslan (2021) found that social exclusion in the school context is negatively related to academic self‐concept and prosocial behavior and positively related to problem behavior. Similarly, Janke et al. (2024) showed that immigrant students experience higher levels of social exclusion depending on their parents' educational level and that this experience weakens their sense of belonging. Furthermore, it was found that the perception of exclusion in the school context is associated with absenteeism and depressive symptoms, and that this relationship emerges through internalized psychological symptoms (Alanko et al. 2025). These findings strongly support the inverse relationship between ostracism and school belonging.

## 3 Psychological Symptoms and School Belonging

Adolescence is a period in which psychological symptoms such as depression, anxiety, and somatization are observed (Zhang et al. 2021), as well as loneliness and behavioral addictions (Kaya et al. 2025; Türk and Yıldırım 2024; Türk et al. 2024a; Türk et al. forthcoming). In the school context, belonging is considered a protective factor that reduces the emergence of such symptoms (Arslan 2021). Belonging is related to students' sense of being accepted at school; this feeling increases psychological resilience and helps them cope with stress (Liu et al. 2025). Especially among adolescents at academic risk, belonging plays a critical role in maintaining psychosocial functioning and psychological well‐being (Arslan and Coşkun 2023).

Research shows that school belonging is inversely related to psychological symptoms such as depression and anxiety and functions as a protective factor (Raniti et al. 2022; Dutcher et al. 2022). Longitudinal data support that low belonging levels in adolescents are associated not only with current psychological distress but also with an increase in depressive symptoms over the long term (Yin et al. 2024). Furthermore, belonging has been found to play a mediating role in explaining the relationship between peer bullying and similar negative school experiences and depression (Jiang and Liang 2021). These findings reveal that belonging serves as a protective buffer and is a critical variable in understanding the impact of contextual factors, such as school climate, on adolescent mental health.

## 4 School Belonging and Meaningful School

When individuals see life as meaningful, their behavioral addictions decrease (Kaya et al. 2024), and their psychological resilience and flexibility increase (Batmaz et al. 2021; Doğrusever et al. 2022; Türk et al. 2024b; Türk et al. 2025b). Besides, especially perceiving school as a meaningful place is related to students viewing their educational life not only as an academic process but also as part of their personal development and life purpose (Arslan and Yıldırım 2021). The perception of meaning at school is a protective factor intertwined with the sense of belonging. Furthermore, school belonging is recognized as a critical resource that supports not only young people's psychosocial adjustment but also their academic achievement, mental health, and overall well‐being (Allen et al. 2018). Research shows that belonging is closely related to perceptions of a meaningful school and that it plays an important role in psychosocial outcomes. For example, Yıldırım et al. (2023) found that adolescents with high belonging experienced school as more meaningful, which was inversely related to internalized and externalized problems such as depression and behavioral problems. Similarly, Arslan and Coşkun (2023) reported that belonging strengthens the perception of meaningfulness in school among academically at‐risk students and that this is linked to psychological well‐being.

## 5 School Belonging and Prosociality

Prosocial behavior encompasses positive social behaviors such as helping others, sharing, and cooperating (Fabes and Spinrad 2019). It has been identified as a factor in successful youth development. During adolescence, such behaviors are seen as a fundamental skill that enhances the quality of peer relationships and strengthens social adjustment (Caprara et al. 2014). Research shows that students with high social support and perceived belonging demonstrate higher classroom and school community participation; this participation also supports prosocial behaviors such as cooperation, sharing, and helping (Hung 2024; Samadieh et al. 2025).

Research indicates that school belonging is an important contextual source for prosocial behaviors in adolescents. For example, Wang et al. (2025) emphasized that belonging plays a parallel mediating role in the relationship between moral identity and prosocial behavior in adolescents. Similarly, Li et al. (2023) revealed the role of peer preferences and social competence in the connection between prosociality and psychological maladjustment, noting that belonging provides a fundamental foundation in this process. All these findings show that belonging not only supports prosocial behaviors but also interacts with peer relationships and moral development.

## 6 Present Study

This study utilized LPA to investigate the associations among ignorance, exclusion, somatization, depression, anxiety, purpose enjoyment, responsible understanding, and prosociality. LPA was chosen for its strength in identifying distinct psychological profiles based on individual variations across these constructs. More than a classification tool, LPA reveals underlying latent structures, presenting evidence about how these variables cluster and interact within individuals. This approach not only deepens theoretical understanding but also supports the development of targeted, profile‐specific interventions.

To guide this inquiry, the current study pursued two central research questions:

RQ1: What latent profiles emerge from the constellation of ignorance, exclusion, somatization, depression, anxiety, purpose enjoyment, responsible understanding, and prosociality?
RQ2: How is school belongingness associated with membership in these latent profiles?

## 7 Methods

### 7.1 Participants

The sample of this study comprised 749 adolescents, including 482 females (64.4%) and 267 males (35.6%). Details regarding the participants are presented in Table 1. The participants’ ages ranged from 13 to 18 years, with a mean of 15.27 years (SD ± 1.25). In LPA research, sample size adequacy was evaluated according to guidelines outlined in the literature. Muthén and Muthén (2002) recommend a minimum sample size of 500 to obtain reliable estimates in complex models. Otherwise, Hair et al. (2010) suggest that the sample size in latent profile analyses should be 5–10 times the number of variables. Consequently, the sample size employed in this study (*n =* 749) can be considered appropriate from an analytical power perspective.

**TABLE 1:** Demographic characteristics of the sample (*n =* 749).

Variables	Options	*n*	*%*
Gender	Female	482	64.4
	Male	267	35.6
Age	*M* = 15.27 Sd = 1.25		
Socioeconomic statuses	Low	85	11.3
	Medium	623	83.2
	High	41	5.5
Discriminated by teachers	Yes	492	65.7
	No	257	34.3
Grade	9^th^ grade	242	32.3
	10^th^ grade	211	28.2
	11^th^ grade	156	20.8
	12^th^ grade	140	18.7

Regarding participants' socioeconomic status (SES), a substantial majority reported medium SES (*n =* 623, 83.2%). A small proportion of participants indicated high SES (*n =* 41, 5.5%), while the remaining participants reported low SES (*n =* 85, 11.3%). Examining the grade levels of adolescents revealed the following distribution: 242 students (32.3%) in ninth grade, 211 students (28.2%) in 10^th^ grade, 156 students (20.8%) in 11^th^ grade, and 140 students (18.7%) in 12^th^ grade. Additionally, 492 adolescents (65.7%) reported experiencing discrimination from their teachers.

The mothers of the participants had varying educational backgrounds, with 195 (26.0%) being illiterate, 283 (37.8%) having completed primary school, 132 (17.6%) graduating from secondary school, 93 (12.4%) finishing high school, and 46 (6.1%) obtaining a bachelor's degree. As for the fathers, 30 (4.0%) were illiterate, 165 (22.0%) had completed primary school, 164 (21.9%) were secondary school graduates, 234 (31.2%) had high school diplomas, and 156 (20.8%) earned bachelor's degrees.

## 8 Measures

### 8.1 School Belongingness Scale (SBS)

Ten items SBS (Arslan and Duru 2017) measured students’ sense of belonging to school. SBS has two subdimensions (school acceptance and school exclusion). Items (e.g., “*I feel that I do not belong to school*” and “*I see myself as a part of this school*”) are rated on a four‐point Likert scale from 1 (*Never*) to 4 (*Always*). A higher score on the scale indicates an increase in the sense of belonging to the school. Scores obtained from the scale range from 10 to 40. Cronbach's alpha coefficient for school acceptance and school exclusion sub‐dimensions was 0.72 and 0.70, respectively. McDonald's ω was respectively 0.72 and 0.70. The current study's confirmatory factor analysis (CFA) indicated that the model fit the data well: *χ*2 / df = 3.82, CFI = 0.93, TLI = 0.90, SRMR = 0.04, RMSEA = 0.04 (Kline 2011).

### 8.2 Ostracism Experience Scale for Adolescents (OES‐A)

Eleven items of the OES‐A (Gilman et al. 2013; Turkish version: Akın et al. 2016) assessed adolescents' perceptions of social ostracism. OES‐A has two subdimensions (ignorance and exclusion). Items (e.g., “*In general, others treat me as if I am invisible*” and “*In general, others invite me to join them for weekend activities, hobbies, or events*”) are rated on a five‐point Likert scale from 1 (*Never*) to 5 (*Always*). The minimum and maximum scores are 11 and 55, respectively. Higher scores indicate higher perceptions of social ostracism. Cronbach's alpha coefficients for the ignorance and exclusion sub‐dimensions were respectively 0.90 and 0.81. McDonald's ω was, 0.90 and 0.85, respectively. The current study's confirmatory factor analysis (CFA) indicated that the model fit the data well: *χ*2 / df = 4.84, CFI = 0.95, TLI = 0.93, SRMR = 0.03, RMSEA = 0.07 (Kline 2011).

### 8.3 Brief Symptom Inventory (BSI‐18)

The eighteen items of the BSI‐18 (Derogatis and Fitzpatrick 2004; Turkish version: Arslan and Yıldırım)^2021^ were used to assess psychological symptoms. BSI‐18 has three subdimensions (depression, anxiety, and somatization). Items (e.g., “*Feelings of worthlessness*,” “*Spells of terror or panic*,” and “*Feeling weak in parts of body*”) are rated on a five‐point Likert scale from 0 (*not at all*) to 4 (*very much*). Each subscale is scored independently. Higher scores indicate greater levels of depression, anxiety, and somatization. An individual's depression, anxiety, and somatization scores are obtained by summing all related items. In the present study, the internal reliability for the depression, anxiety, and somatization subscales was *α* = 0.84, *α* = 0.83, and *α* = 0.82, respectively. McDonald's ω was 0.84 for depression, 0.83 for anxiety, and 0.82 for somatization, respectively. The current study's confirmatory factor analysis (CFA) indicated that the model fit the data well: *χ*2 / df = 3.17, CFI = 0.94, TLI = 0.93, SRMR = 0.05, RMSEA = 0.05 (Kline 2011).

### 8.4 Meaningful School Questionnaire (MSQ)

Ten items’ MSQ (Arslan and Yıldırım 2021) assessed adolescents' meaning in life in the school context. MSQ has two subdimensions (responsible understanding and purpose enjoyment). Items (e.g., “*In general, I feel good about my school life*” and “*I take full responsibility for my academic success*”) are rated on a five‐point Likert scale from 1 (*strongly disagree*) to 5 (*strongly agree*). The minimum and maximum scores are 10 and 50, respectively. Higher scores indicate higher perceptions of meaning in life in the school context. Cronbach's alpha coefficient for the understanding and purpose enjoyment sub‐dimensions was 0.71 and 0.82, respectively. McDonald's ω was respectively 0.69 and 0.82. The current study's confirmatory factor analysis (CFA) indicated that the model fit the data well: *χ*2 / df = 2.90, CFI = 0.96, TLI = 0.95, SRMR = 0.03, RMSEA = 0.05 (Kline, 2011).

### 8.5 Student Prosociality Scale (SPS)

Four items’ SPS (Renshaw 2014; Turkish version: Arslan and Tanhan, 2019) assessed adolescents' prosocial behavior in school settings. Items (e.g., “*I help other kids who seem to be having a hard time, I am kind to my friends at school*”) are rated on a four‐point Likert scale from 1 (*almost never*) to 4 (*almost always*). The minimum and maximum scores are 4 and 16, respectively. Higher scores indicate higher student prosociality in the school context. In the present study, Cronbach's alpha coefficient was calculated as .70, and McDonald's ω was .70. The current study's confirmatory factor analysis (CFA) indicated that the model fit the data well: *χ*2 / df = 3.67, CFI = 0.99, TLI = 0.97, SRMR = 0.02, RMSEA = 0.04 (Kline, 2011).

### 8.6 Procedure

The present study employed convenience sampling. The research data were gathered from a large sample of high school students over the 2023–2024 academic year. Before data collection, school administrators and the school's psychological counsellor were informed about the research purpose. Students who volunteered to participate were required to have their parents sign an informed consent form. The researcher entered each classroom and explained the study's purpose to the students. Participants were reminded of their right to withdraw at any time, that their data would remain confidential, and that they should not share personal information on forms. Since students' personal phone use was prohibited at the schools where the research was conducted, all data were collected face‐to‐face using paper‐based surveys. The surveys take approximately 15 to 20 min to complete.

The study included high school students aged 13–18 years who were willing and emotionally capable of participating in social and emotional assessments. Participants were excluded if they met any of the following conditions: (a) had a previous psychiatric diagnosis, (b) had chronic absenteeism, (c) deliberately completed assessment scales incompletely or carelessly, or (d) wished to withdraw from the research at any stage. Information regarding psychiatric diagnosis history and chronic absenteeism was obtained through self‐report items included in the demographic information form completed by participants. This research was approved by the ethics committee at Siirt University (reference number: 6034), and all stages of the study were conducted in accordance with the Declaration of Helsinki.

### 8.7 Statistical Analyses

First, the variables' assumptions and descriptive statistics were assessed before data analysis. Using a face‐to‐face data collection method with paper‐based surveys results in some forms being found to be incomplete or incorrectly filled out. Following data screening, 38 forms with substantial incompleteness (>30% missing per case) were removed. Subsequently, missing data analysis revealed that missing values across all study variables ranged from 0.1% to 2%, well below the 5% threshold generally considered unlikely to bias results (Schafer 1999). Little's MCAR test (*χ* ^2^ = 4051.99, df = 3486, *p* < 0.001) suggested data were not missing completely at random; however, given the minimal amount of missing data (<5%), analyses proceeded under the missing at random (MAR) assumption, which is appropriate for Full Information Maximum Likelihood (FIML) estimation (Enders 2010).

All analyses were conducted using Mplus with FIML estimation to handle missing data. FIML uses all available information from each case and provides unbiased parameter estimates under MAR (Enders 2010; Graham 2009). Composite scores were computed from item‐level data, and LPA was conducted using Maximum Likelihood estimation with Robust standard errors (MLR), which appropriately accounts for missing data uncertainty.

Multivariate outliers were identified using Mahalanobis distance (*χ* ^2^ critical value = 27.88, df = 9, *p* < 0.001) based on nine variables: ignorance, exclusion, somatization, depression, anxiety, purpose enjoyment, responsible understanding, prosociality, and school belongingness. Thirteen cases (1.7% of the original sample, *N =* 762) exceeded this threshold. Visual inspection of item‐level responses revealed that all 13 cases exhibited straight‐lining behavior (e.g., responding “5, 5, 5, 5, 5” or “1, 1, 1, 1, 1” across entire scales), consistent with careless responding (Curran 2016; Meade and Craig 2012) rather than genuine psychological extremes. These cases did not form a clinically coherent subgroup. Sensitivity analyses confirmed minimal impact of their removal on results (correlation differences < 0.05, identical LPA solutions). Following outlier removal, analyses were conducted with *N =* 749.

Second, to validate the structural integrity of each measurement tool in the study, we employed confirmatory factor analysis (CFA) with the maximum likelihood (ML) estimation method. To evaluate the model's fit, we used four key fit indices: the Comparative Fit Index (CFI), Tucker‐Lewis Index (TLI), Root Mean Squared Error of Approximation (RMSEA), and Standardized Root Mean Square Residual (SRMR), following the methodological guidelines proposed by Jackson et al. (2009). The model fit was considered adequate when the Comparative Fit Index (CFI) and Tucker‐Lewis Index (TLI) values exceeded 0.90, the Root Mean Square Error of Approximation (RMSEA) values were less than 0.08, and the chi‐square to degrees of freedom (*χ* ^2^/df) ratios were below 5 (Kline 2011).

**FIGURE 1:**
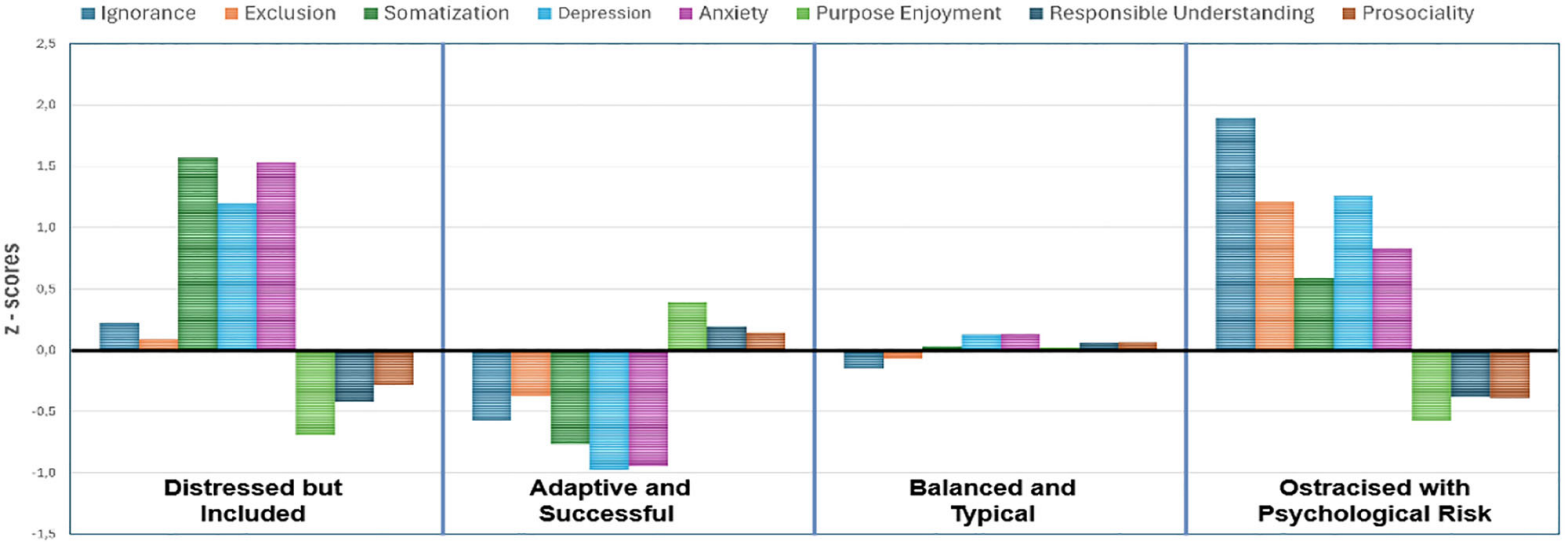
Four‐profile solution bar graph.

Third, LPA was conducted to identify the profiles within the data set. LPA was conducted using eight indicators: subscale scores for ignorance, exclusion, somatization, depression, anxiety, purpose enjoyment, responsible understanding, and prosociality. Raw scores (i.e., the sum of item responses for each subscale) were used in the LPA estimation. Moreover, to facilitate readers’ interpretation of the profiles, z‐scores were computed for visualization purposes and used to generate the profile plot presented in Figure 1. ML estimation with robust standard errors (MLR) was employed to handle potential non‐normality in the data. To enhance the robustness of our statistical analysis, we employed a comprehensive model estimation approach using 1000 random start values, conducting 500 iterations and 250 final optimizations (Hipp and Bauer 2006; Muthén and Muthén 2017). Notably, all models converged successfully to a consistent and replicable solution. The LPA was carried out in three steps:

1. Determination of latent profiles

Models with one and five profiles were tested to determine the optimal number of profiles. To assess how well the models fit the data, we used three criteria: the Akaike Information Criterion (AIC) (Akaike 1974), the Bayesian Information Criterion (BIC) (Schwarz 1978), and the sample‐size‐adjusted BIC (SABIC) (Sclove 1987). Lower values of these criteria indicate a better fit (Ferguson et al. 2020). To determine the optimal number of profiles, we employed the Lo‐Mendell‐Rubin likelihood ratio test (Lo et al. 2001). This test compares models with different numbers of profiles. The best‐fitting model was selected using the bootstrapped likelihood ratio test (BLRT). We also examined the entropy values. Higher entropy values (above 0.8) indicate a better fit (Muthén 2004). We ensured that the smallest profile accounted for at least 5% of the sample, as recommended by Marsh et al. (2009). Finally, z‐scores were calculated for each profile variable, and the data were standardized to facilitate comparison. These z‐scores were visualized using a bar graph, with each profile represented by a different color to improve interpretability and show the standardized means of the key variables.

2. Comparison of profiles:

Once the number of profiles was established, one‐way ANOVA was used to determine whether the scores for students’ ostracism (ignorance, exclusion), emotional problems (somatization, depression, anxiety), meaningful school life (purpose, enjoyment, responsible understanding), and prosociality varied among the different profiles. Post hoc analyses, using the Tukey HSD and Games‐Howell tests, were performed to compare group means. Additionally, effect sizes were calculated based on the ANOVA results. Eta‐squared (η^2^) values of 0.01 are interpreted as small, 0.06 as medium, and 0.14 as large effect sizes (Green and Salkind 2005).

3. Regression analysis on profile membership

To examine the associations of school belongingness (Model 1) and teacher discrimination (Model 2) with profile membership, we employed a multinomial logistic regression approach using the AUXILIARY command in Mplus (Nylund‐Gibson et al. 2019). Two separate models were estimated: the first included school belongingness (continuous variable) as the predictor, while the second examined teacher discrimination (dichotomous variable: 0 = no, 1 = yes). In the AUXILIARY command, both analyses employed the R3STEP approach, which accounts for classification uncertainty from the LPA when testing associations with covariates (Asparouhov and Muthén 2014). Overall, the most suitable group (Profile 2) was selected as the reference group, and comparisons were subsequently conducted.

Data analyses used IBM SPSS 26 for internal consistency coefficients, descriptive statistics, and analysis of variance (ANOVA) tests. Bivariate correlations, confirmatory factor analysis, LPA, and multinomial logistic regression analyses were conducted using MPlus 8.3.

## 9 Results

### 9.1 Descriptive Statistics

Table 2 presents the mean, standard deviation, skewness, and kurtosis values obtained for the variables utilized in the study. Additionally, the bivariate correlations between the variables are provided, and the results show that the coefficients are statistically significant.

**TABLE 2:** Descriptive statistics and bivariate correlations among variables (*N =* 749).

Variable	1.	2.	3.	4.	5.	6.	7.	8.	9.
1. Ignorance	1								
2. Exclusion	0.50^**^	1							
3. Somatization	0.34^**^	0.18^**^	1						
4. Depression	0.54^**^	0.32^**^	0.54^**^	1					
5. Anxiety	0.41^**^	0.20^**^	0.68^**^	0.73^**^	1				
6. Purposeful enjoyment	−0.23^**^	−0.19^**^	−0.25^**^	−0.38^**^	−0.28^**^	1			
7. Responsible understanding	−0.22^**^	−0.20^**^	−0.16^**^	−0.26^**^	−0.17^**^	0.59^**^	1		
8. Prosociality	−0.13^**^	−0.09^*^	−0.11^**^	−0.14^**^	−0.11^**^	0.36^**^	0.28^**^	1	
9. School belongingness	−0.45^**^	−0.39^**^	−0.27^**^	−0.40^**^	−0.32^**^	0.47^**^	0.38^**^	0.25^**^	1
Mean	8.78	16.90	7.36	10.43	9.40	16.75	19.37	13.80	29.40
Std. Deviation	4.25	5.87	5.37	6.28	5.59	4.72	3.53	2.04	5.25
Skewness	1.21	0.25	0.70	0.20	0.39	−0.39	−0.91	−1.39	−0.42
Kurtosis	0.98	−0.72	−0.05	−0.87	−0.57	−0.57	1.06	2.60	−0.09

*Note*: ^**^ *p* < 0.001; ^*^ *p* < 0.05.

### 9.2 LPA

To identify distinct subgroups of participants, a series of latent profile models ranging from one to five profiles was compared. Table 3 presents the fit indices for each model. While the five‐profile solution yielded the lowest AIC, BIC, and SABIC values, closer inspection revealed that the LMR test failed to reach statistical significance (*p* = 0.140), suggesting that the fifth profile did not substantially improve model fit. The entropy value of 0.817 for this model, although acceptable, could not compensate for the non‐significant LMR result.

**TABLE 3:** Fit indices for latent profile models.

Model	free param.	AIC	BIC	SABIC	LL	Entropy	LMR (p)	BLRT	Profile Sizes (%)
1	16	34278.366	34352.265	34301.459	−17123.183	—	—	—	—
2	25	33165.151	33280.619	33201.234	−16557.575	0.830	.000	.000	34.0 / 66.0
3	34	32846.075	33003.112	32895.149	−16389.038	0.799	.001	.000	35.4 / 43.3 / 21.3
**4**	**43**	**32651.278**	**32849.884**	**32713.342**	**−16282.639**	**0.828**	**.051**	**.000**	**11.1 / 33.8 / 43.3 / 11.9**
5	52	32480.383	32720.558	32555.438	−16188.192	0.817	.140	.000	11.7 / 11.0 / 34.0 / 31.1 / 12.2

*Note*: Boldface indicates the best‐fitting model based on fit indices.

Abbreviations: AIC = Akaike information criterion; BIC = Bayesian information criterion; SABIC = sample‐size adjusted BIC; LL = log‐likelihood; LMR = Lo‐Mendell‐Rubin likelihood ratio test; BLRT = bootstrap likelihood ratio test.

Beyond statistical criteria, theoretical interpretability was also considered in model selection. When the five‐profile model was examined, the additional profile did not represent a conceptually distinct subgroup. Instead, it appeared as a smaller version of an already existing profile. For instance, both profiles (Profile 3 and Profile 4) exhibited similarly high purpose enjoyment, responsible understanding, and prosociality, and low somatization, depression, and anxiety, differing only in the degree of ostracism (minimal in Profile 4, moderate in Profile 3) rather than in their overall adaptive configuration. Adding such a profile would increase model complexity without providing new insights into adolescent heterogeneity. In contrast, each of the four‐profile models had an interpretable pattern: adolescents who were distressed but socially included, those who were adaptive and successful in the group, those who showed balanced and typical characteristics, and those who experienced ostracism alongside psychological risk. This solution was chosen because it offers both statistical and theoretical clarity (Marsh et al. 2009).

A comparison between the three‐profile and four‐profile models supported the four‐profile model. The four‐profile model demonstrated lower AIC, BIC, and SABIC values than the three‐profile model. The LMR test approached conventional significance levels (*p* = 0.051), while the BLRT yielded a significant result (*p* < 0.001). Given that BLRT is generally considered a more reliable indicator of model fit than LMR and considering the overall pattern of fit indices, the four‐profile solution was deemed preferable. Entropy for this model (0.828), indicating adequate classification precision. This finding was corroborated by average posterior probabilities, which ranged from 0.89 to 0.92 across the four profiles, above the commonly applied 0.70 criterion. The smallest profile consisted of 11.3% of the sample, meeting the recommended minimum threshold.

After determining the number of profiles, z‐scores were calculated using the means across the 4 model solutions; Figure 1 illustrates the bar graph representation with four distinct profiles based on the following variables: ostracism (ignorance, exclusion), emotional problems (somatization, depression, anxiety), meaningful school life (purpose, enjoyment, responsible understanding), and prosociality.

Profile 1 comprises 83 students (11.1% of the participants). This profile demonstrates high scores on emotional problems (somatization, depression, and anxiety) while showing below‐average levels of meaningful school life and prosociality. Ostracism (ignorance, exclusion) remains close to average. This pattern reflects adolescents who experience internalized difficulties despite being socially included. Accordingly, this group was named “Distressed but Included Group.”

Profile 2 comprises 253 students (33.8% of participants), forming the second‐largest subgroup. Adolescents in this profile exhibit low levels of ostracism and emotional problems alongside above‐average meaningful school life and prosociality scores. This profile reflects well‐adjusted individuals who thrive socially and academically. Consequently, this group has been named the “Adaptive and Successful Group.”

Profile 3 comprises 324 students (43.3% of the participants), representing the largest profile group. In this profile, all variables demonstrate near‐average trajectories, indicating a normative pattern with neither strengths nor weaknesses. These adolescents can cope with the typical developmental challenges they face in their lives without experiencing noteworthy emotional problems or ostracism. Consequently, this group has been named the “Balanced and Typical Group.”

Profile 4 comprised 89 students (11.9% of participants). Although this profile shares a similar sample size and percentage with Profile 1, it is distinguished from all other profiles (Profiles 1, 2, and 3) by markedly high levels of ostracism (both ignorance and exclusion) and emotional difficulties, particularly depression. The group appears to be experiencing important challenges and may require targeted interventions. In other words, this profile represents individuals encountering substantial emotional difficulties, especially depression, while simultaneously reporting low satisfaction with school life. Consequently, this group has been named the “ *Ostracized with Psychological Risk Group*.”

### 9.3 Examining the Differences Among the Latent Profiles

After determining the optimal number of latent profiles for students, the latent profiles for ostracism (ignorance, exclusion), emotional problems (somatization, depression, anxiety), meaningful school (purpose, enjoyment, responsible understanding), and prosociality to change were compared using a one‐way ANOVA. According to Table 4, all latent profiles have significant F values.

**TABLE 4:** ANOVA results examined the differences across the four latent profiles.

Variables	Profile 1 (*n =* 83) M(SD)	Profile 2 (*n =* 253) M(SD)	Profile 3 (*n =* 324) M(SD)	Profile 4 (*n =* 89) M(SD)	*F* (3, 745)	η^2^
Ignorance	9.61 (2.99)^b^	6.39 (2.24)^d^	8.22 (3.06)^c^	17.07 (3.16)^a^	324.88^∗∗∗^	(0.57)
Exclusion	17.22 (6.31)^b^	14.57 (4.70)^c^	16.64 (5.41)^b^	24.01 (4.05)^a^	74.26^∗∗∗^	(0.23)
Somatization	15.89 (4.37)^a^	3.24 (2.87)^d^	7.52 (3.95)^c^	10.48 (4.49)^b^	266.73^∗∗∗^	(0.52)
Depression	17.78 (3.75)^a^	4.00 (2.94)^d^	11.35 (3.75)^c^	18.26 (3.47)^a^	573.71^∗∗∗^	(0.70)
Anxiety	17.94 (3.42)^a^	3.96 (2.43)^d^	10.22 (3.39)^c^	13.96 (4.19)^b^	502.59^∗∗∗^	(0.67)
Purpose enjoyment	13.45 (4.43)^c^	18.68 (4.31)^a^	16.96 (4.21)^b^	13.78 (4.79)^c^	46.58^∗∗∗^	(0.16)
Responsible Understanding	17.29 (3.74)^b^	20.27 (3.44)^a^	19.65 (3.13)^a^	17.63 (3.79)^b^	24.98^∗∗∗^	(0.09)
Prosociality	13.20 (2.33)^b^	14.12 (2.87)^a^	13.93 (1.74)^a^	12.98 (2.40)^b^	9.97^∗∗∗^	(0.04)

*Note*: ^***^ *p* < 0.001. Means with different superscript letters within each row differ significantly at *p* < 0.05 based on post‐hoc tests (Tukey HSD for Purpose Enjoyment and Responsible Understanding; Games‐Howell for other variables due to violation of homogeneity of variance).

Post‐hoc analyses revealed several notable patterns. Students in Profile 2 (Adaptive and Successful) consistently demonstrated the most favorable outcomes, scoring significantly higher on meaningful school life and prosociality, while reporting lower levels of ostracism and emotional problems than students in other profiles. Profile 3 (Balanced and Typical) generally fell between the extremes, showing moderate levels across most variables. In contrast, Profile 4 (Ostracized with Psychological Risk) exhibited the highest levels of ostracism alongside high depression scores, combined with diminished meaningful school life and prosociality. Profile 1 (Distressed but Included), though not experiencing high ostracism, reported the highest somatization and anxiety levels among all groups, coupled with reduced school engagement.

Lastly, the effect sizes for these group differences were substantial, with η^2^ values ranging from 0.04 to 0.70. Most variables demonstrated large effect sizes (η^2^ > 0.14), with particularly strong effects observed for emotional problems, indicating considerable differentiation among the four profiles in their psychosocial functioning.

### 9.4 Multinomial Logistic Regression

In the final stage of the analysis, to examine whether school belongingness and teacher discrimination predicted latent profile membership, two separate multinomial logistic regression analyses were conducted. Profile 2 is set as the reference category. The findings from the multinomial logistic regression analysis are presented in Table 5.

**TABLE 5:** Multinomial logistic regression results.

	Profile 1 vs. Profile 2		Profile 3 vs. Profile 2		Profile 4 vs. Profile 2	
	OR	95% CI	OR	95% CI	OR	95% CI
Model 1: School belongingness	1.383^***^	[1.28, 1.49]	1.184^***^	[1.11, 1.26]	1.116^**^	[1.03, 1.20]
Model 2: Teacher Discrimination (Yes vs. No)	3.377^*^	[1.81, 6.29]	1.716	[0.91, 3.22]	0.840	[0.34, 2.08]

*Note*: ^***^ *p* < 0.001, ^**^ *p* < 0.01, ^*^ *p* < 0.05. Reference group = Profile 2 (Adaptive and Successful). Model 1 and Model 2 represent separate analyses.

Abbreviations: CI = Confidence Interval; OR = Odds Ratio.

According to Table 5, multinomial logistic regression examined the associations between school belongingness (Model 1) and latent profile membership. Using Profile 2 (Adaptive and Successful) as the reference category, results showed that low school belongingness significantly increased students' likelihood of membership in less adaptive profiles. Specifically, for each one‐unit decrease in sense of belongingness, students had a 1.38‐fold increased likelihood of being in Profile 1 (Distressed but Included; OR = 1.383, 95% CI [1.28, 1.49], *p* < 0.001). Students were 1.18 times more likely to be in Profile 3 (Balanced and Typical; OR = 1.184, 95% CI [1.11, 1.26], *p* < 0.001) and 1.12 times more likely to be in Profile 4 (ostracized with Psychological Risk; OR = 1.116, 95% CI [1.03, 1.20], *p* < 0.01) compared to Profile 2.

The second model examined teacher discrimination (Model 2) as a predictor of profile membership. Students who reported experiencing discrimination from their teachers had significantly higher odds of belonging to Profile 1 compared to Profile 2 (OR = 3.377, 95% CI [1.81, 6.29], *p* < 0.05). This indicates that students experiencing teacher discrimination were approximately 3.4 times more likely to belong to the profile characterized by serious emotional problems despite relatively intact peer relationships. In contrast, teacher discrimination did not significantly predict membership in Profile 3 (OR = 1.716, 95% CI [0.91, 3.22], *p* = 0.194) or Profile 4 (OR = 0.840, 95% CI [0.34, 2.08], *p* = 0.680).

These findings suggest that school belonging is significantly associated with the likelihood of membership in different profiles and that teacher discrimination is a specific risk factor for Profile 1, characterized by emotional difficulties in the absence of peer rejection.

## 10 Discussion

In this study, LPA was used to understand adolescents' psychosocial adjustment and school experiences more holistically. The research findings show that students cannot be reduced to mere “good/bad” categories; rather, distinct profiles emerge based on different patterns of psychological and social variables. In particular, the co‐occurrence of high ignorance, exclusion, depression, anxiety, and somatization with low school belonging and low prosocial behavior in the “Ostracized with Psychological Risk Profile,” and similarly, high depression, anxiety, and somatization and an average level of ostracism in “the Distressed but Included Profile” indicates that these students require multi‐layered support. Similarly, LPA revealed that prosocial behavior was low in profiles dominated by emotional and behavioral difficulties and that these students showed more negative outcomes in terms of subjective well‐being and academic adjustment. Arbel et al. (2023) also found that adolescents can be divided into distinct profiles based on prosocial behavior and peer relationships, and that these profiles differ in terms of psychological adjustment.

A positive school environment and strong teacher relationships support students in various aspects. For example, the literature indicates that a harmonious school environment characterized by strong teacher‐student relationships enhances adolescents' adaptive capacity by reducing depression, anxiety, and stress (Zhang et al. 2019). Similarly, it was observed that the adolescents had low levels of exclusion and emotional problems and high levels of adaptation, socialization, and meaningful school scores, such as the characteristics of profile 2, “*adaptive and successful profile*.” However, profile 3, “balanced and typical profile,” represented the largest and most balanced student group in the study. Considering the characteristics of the adolescents in this group, it is possible to argue that they can cope with individual developmental crises and the exclusionary behaviors they encounter in a balanced manner. Indeed, in latent profile studies addressing the exclusion of adolescents by their teachers, parents, and peers, it was observed that low‐ and medium‐risk profiles exhibited more balanced levels of negative characteristics (e.g., suicide, social and emotional loneliness, and internalizing and externalizing problems) (Kiuru et al. 2024; Tan et al. 2025).

Multinomial logistic regression analyses revealed that school belonging and teacher discrimination were significantly associated with membership in these profiles. As belonging increases, students' likelihood of falling into healthy and well‐adjusted profiles, such as adaptive and successful, rises. This finding shows that school belonging is not only a fundamental structure that guides students' institutional commitment but also their psychological functioning and social behavior. Previous studies have similarly shown that school belonging is a protective factor that reduces depression and anxiety, increases psychological resilience, and supports overall mental health (Allen et al. 2024). Furthermore, Yin and colleagues (2024) revealed that school belonging is a critical mediating mechanism in the relationship between negative school climate and depressive symptoms, while Pastor and colleagues (2025) emphasized that belonging is a powerful protective factor that supports mental health for young people. On the other hand, being exposed to teacher discrimination can have devastating consequences for students. For example, Jiang et al. (2023) reported that adolescents experiencing teacher discrimination developed suicidal thoughts. Similarly, Maene et al. (2022) found that disadvantaged students (e.g., immigrants or victims of racism) were most likely to develop emotional problems (e.g., depression) when they were exposed to teacher discrimination.

The prevalence of these variables together in profiles characterized by meaningful school life and high levels of prosocial behavior demonstrates that school is not merely an academic institution but also an environment where social and psychological functions develop. Students' perception of their school experiences not as a passive obligation but as a purposeful, participatory process integrated with social relationships stands out as a defining feature of harmonious profiles. Previous studies have also shown that school belonging makes education more meaningful, positively affecting both academic adjustment and mental health (Arslan and Yıldırım 2021; Yıldırım et al. 2023; Datu and Valdez 2019). Furthermore, the parallel increase in prosocial behavior with belonging is consistent with findings showing that students' social participation and peer relationships are strengthened (Wang et al. 2025; Hung 2024; Samadieh et al. 2025). These results are also consistent with previous reviews and empirical findings on school belonging (Slaten et al. 2016; Ahmadi et al. 2020; Vaz et al. 2015).

## 11 Implications

These results demonstrate the theoretical contribution of examining the link between psychosocial adjustment and school belonging in adolescence using a profile‐based approach. Going beyond the limitations of analyses based on individual variables to reveal the unique patterns of different student groups contributes to both theoretical knowledge and applied educational policies. It is important for educational institutions to develop profile‐based, targeted support models, especially for high‐risk groups, rather than relying on general interventions. Practices such as directing students in the Psychological Risk Group to both psychological support and belonging‐enhancing programs and organizing activities that reinforce community spirit can be beneficial in this regard. This approach is consistent with previous findings emphasizing the supportive effect of school climate on mental health through belonging (Chen et al. 2025).

## 12 Limitations

This study has some limitations. First, the data were collected within a single cultural and regional context. This may limit the generalizability of the findings to different cultures and education systems. Second, the research uses a cross‐sectional design, which does not provide causal evidence of the relationships between variables. Third, the measurement tools used are self‐report scales, which may introduce limitations such as social desirability or recall bias. Finally, only specific psychosocial and school‐based variables were considered in the analyses. The exclusion of other factors, such as family support, socioeconomic status, or teacher‐student relationships, may be considered a limitation that could lead to a more restricted interpretation of the profiles.

There are some limitations to the method that should be noted. Firstly, the missing values data were handled using FIML. Although Enders (2010) and Graham (2009) stated that this method preserves statistical power, subsequent studies may consider comparing the values obtained from multiple imputation of missing data with those obtained from FIML to verify the findings. Second, conducting analyses based solely on participants' self‐reports may lead to social desirability bias. Future research would benefit from incorporating objective outcomes into the analysis, such as teachers' views of their students, parents' emotional feedback about their children, students' actual grades, absences, and disciplinary incidents. Third, cross‐sectional studies do not demonstrate changes over time in the relationships between school belonging and profile membership. That is why it prevents causal conclusions. Furthermore, the direction of the relationship between school belonging and profile membership cannot be determined; it is equally possible that low school belonging contributes to psychological difficulties or that psychological difficulties lead to a reduced sense of school belonging. Prospective longitudinal studies using LPA will yield valuable results by demonstrating how school belonging changes across students' developmental stages and how it transitions from maladaptive to adaptive profiles over time. In summary, future longitudinal studies are needed to uncover temporal and causal relationships.

## 13 Conclusion

This study examined adolescents' school belonging and psychosocial adjustment using LPA and identified different student profiles. The findings revealed that students in risk groups require multifaceted support. Overall, it was concluded that school belonging plays a central role in students' psychological and social adjustment. Finally, school belonging and teacher discrimination were found to be predictors of emerging profiles.

## Author Contributions

**Caner Doğrusever**: study conception and design, data collection, analysis, drafting of manuscript, statistical expertise, supervision, editing, administrative/technical/material support, writing – original draft preparation. **Hacer Yıldırım Kurtuluş**: writing – original draft preparation. **Alican Kaya**: study conception and design, data collection, analysis, drafting of manuscript, statistical expertise, supervision, editing, administrative/technical/material support, writing – original draft preparation. **Nuri Türk**: study conception and design, data collection, analysis, drafting of manuscript, statistical expertise, supervision, editing, administrative/technical/material support, writing – original draft preparation. **Murat Yıldırım**: writing – original draft preparation, writing – review and editing.

## Funding

The authors have nothing to report.

## Ethics Statement

Ethical approval was granted by the Research Ethics Committee of Siirt University (Report No: 6034).

## Consent

Consent was obtained from all participants included in the study.

## Conflicts of Interest

The authors declare no conflicts of interest.

## Data Availability

The data supporting this study's findings are available from the corresponding author upon reasonable request. The data were anonymized, ensuring that there was no breach of privacy. It will be shared in a manner that respects ethical protocols and data protection regulations. The dataset will be accessible only for academic purposes, and any use of the data will recognize the original study and maintain the confidentiality of the participants.
